# Woody species composition and community types of Hangadi Watershed, Guji Zone, Ethiopia

**DOI:** 10.1186/s12862-021-01949-9

**Published:** 2021-12-28

**Authors:** Berhanu Tamiru, Teshome Soromessa, Bikila Warkineh, Gudina Legesse, Merga Belina

**Affiliations:** 1 Center of Enviornmental Sciences, Addis Ababa University, Addis Ababa, Ethiopia; 2 Department of Plant Biology and Biodiversity Management, Addis Ababa University, Addis Ababa, Ethiopia; 3 Statitics Department, Addis Ababa University, Addis Ababa, Ethiopia

**Keywords:** Hangadi, Biodiversity, Plant community, Cluster, Ordination

## Abstract

**Background:**

Hangadi watershed is endowed with the only virgin forest in Odo shakisso harbouring high biodiversity, but it has been suffered from anthropogenic activities. This study was conducted to investigate composition and community diversity of woody species in restoration for posterity. Satellite images of 1988, 2008, and 2018 were used to classify and analyse trends of deforestation. For both woody species and topsoil (0–30 cm), 20 m × 20 m, 100 plots laid at every 300 m along line transects, 1 km apart from each other. In each sample plot, woody species ≥ 3 m were counted, Shannon–wiener diversity index, cluster analysis and ordination were computed.

**Results:**

Agroforestry is found to be the dominant land use/land cover class followed by forest and cultivated land. A total of 61 woody species belonging to 34 families; 8.2% of the species were endemic to Ethiopia. The highest number of species was recorded from families Euphorbiaceae and Rubiaceae (5 species, 8.2%), Rutaceae, Celastraceae, and Oleaceae (3 species, 5.08%) followed by Flacourtiaceae, Meliaceae, Araliaceaae, Myrsinaceae, Moraceae, Boraginaceae, Asteraceae, Spontaceae, Lauraceae and Sapindaceae (2 species each). Four woody plant communities were identified using free statistical software R version 3.1.1. The canonical correspondence analysis result showed that EC, pH, OM, altitude, C:N, CEC, sand, silt, AvP, and TN significantly affected species distribution in the watershed.

**Conclusion:**

Local people involved in cutting mature woody species for timber production, making farm implements and, cultivated land expansion. Protection of woody species diversity of forest and coffee systems with low biodiversity value conservation concepts are recommended to be executed jointly by local people and stakeholders.

## Background

Biodiversity is defined: living organisms’ variability both the terrestrial, marine, and the genes contained and ecosystems they form covering the variety of life on different scales [25]. Variety of plant and animal species, ecosystems, and genes within those species are mentioned as biodiversity. It is understood as a critical factor for sustainability of life. The biodiversity losses are attributed to both direct and indirect drivers [38]. According to [16], biodiversity in general provides supporting (nutrient cycling, primary production), provision (food, timber, fuel, freshwater), and regulating (climate and water regulation) and cultural (spiritual experience, recreation, education) are ecosystem services for human wellbeing.

Population increment has put significant pressure on global biodiversity through deforestation, habitat fragmentation, and overexploitation of species [6]. Land conversion for agriculture and agricultural intensification, logging, fuelwood collection, cattle grazing, and commercial forest management are direct causes of the decline of woody species. Of which habitat loss, over-harvesting, and climate change are the major ones [22, 24]. The indirect drivers such as population growth, economic activities, certain property rights, policies, socio-cultural factors, and markets influence local people’s ability to maintain woody plants and associated benefits [46].

Forest cover in Ethiopia declined from 15.11 million hectare in 1990 to 12.9 million hectare in 2010, during which 18.66% of the forest cover was deforested within those 20 years. As a result, nearly 141,000 hectare was destroyed every year [9, 22]. The accelerated conversion of forests to agricultural land-use types and overutilization of forest resources to satisfy the increasing population’s food and energy requirements are significant environmental concerns [14]. Patterns of deforestation will soon decimate the remaining forests unless suitable interventions are designed. The causes of deforestation are closely linked with the vicious circle of mutually reinforcing factors such as poverty, population growth, poor economic growth and climate change. Such deforestation results in loss of biodiversity, drought, ecological imbalance, and environmental degradation [19].

Growing literatures such as [24, 62] found out that local people use forest to obtain fire and construction wood, and farm tools, as well as for livestock grazing, medicine and spices. Species including important pole and timber appeared to be overharvested in forests preferring agroforestry to forest land uses for sustainability of forests. However, [15, 30, 51] summarized in coffee agroforestry, slashing of vegetation and related modifications of forest microclimate have a strong impact on biodiversity values. As land use converted from forest to agroforestry, and agroforestry to cultivated land, there is a significant reduction in woody species diversity, composition and population structures [28]. It triggers for a conservation concept both protection of the original woody species diversity of forest and profitable use of coffee systems having lower biodiversity value.

Guji zone, the study area was known by pastoral means of existence, suggesting it was covered with forest. As per the information from district bureau of agriculture and key informants, two decades ago, local community and some investors started coffee cultivation as agroforestry. It was done at the expense of the only virgin forest in the district without considering its impacts on forests’ climate and ecological potential. Analysis of floristic composition and diversity is needed to ensure vegetation conservation. No effort has been made to undertake a quantitative analysis of the woody species communities essential to document the remaining vegetation resources for restoration for posterity in the watershed. Hence, data on woody species composition and community are lacking in Hangadi watershed. The current study seeks to help fulfill this knowledge gap. It is believed to contribute to the efforts made in the development of a sound management plan for the effective conservation of forest resources in the study area. Therefore, this study's objectives were to.

1. Assess land use and land cover dynamics
2. Determine the floristic composition and diversity of woody species in the Hangadi watershed and
3. Identify edaphic and topographic factors responsible for woody plant community and distribution patterns along the gradients.

## Methods

### Study area

**Fig. 1:**
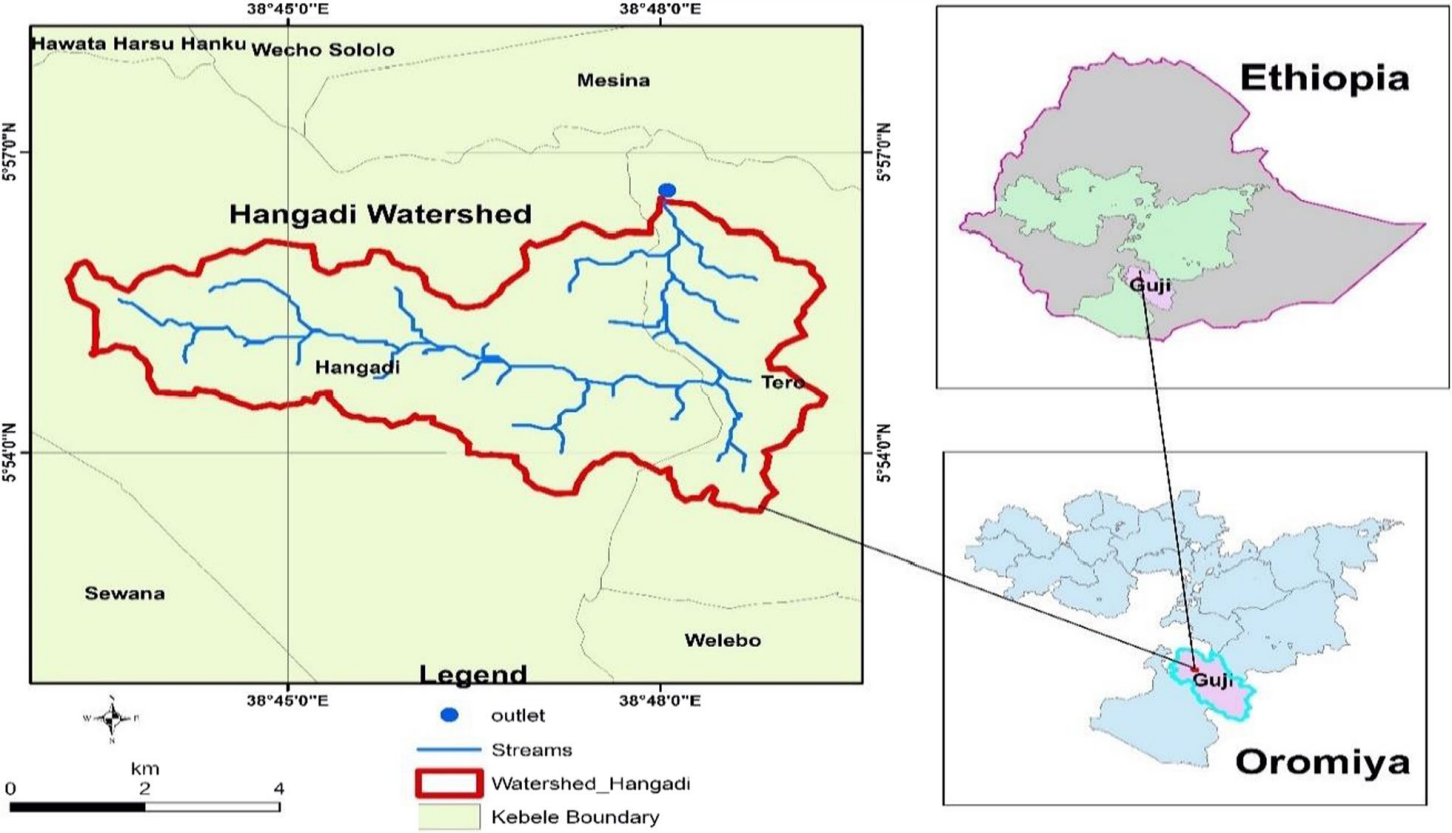
Map of Ethiopia showing the study watershed

The study was conducted in Hangadi Watershed, Odo-Shakiso district of Guji administrative zone, Oromia National Regional State, Ethiopia (Fig. 1). Guji zone is bordered on the south by Borena zone, on west by Southern Nations, Nationalities, and peoples Region, on the North by Bale zone and on the east by Somale region. The study area is situated 550 km south of Addis Ababa at 38°10ʹE, 5°34 Nʹ and bordered in the south, west northeast, north and east by Dawa River, separates it from Arero, Bule Hora, Uraga and Bore, Adola and Wadera, and Liben districts, respectively as per the information from local informants and district administration offices. According to the projection of CSA [12], district’s population is 268,630 (148,724 men and 119,906 women) with lowland 15%, midland 20% and highland 65%. The district comprises an area coverage of 4165.62 km^2^ with a density of 59.3 people per km^2^. The study area comprises three land use types (forest, agroforestry and cultivated land).

**Fig. 2:**
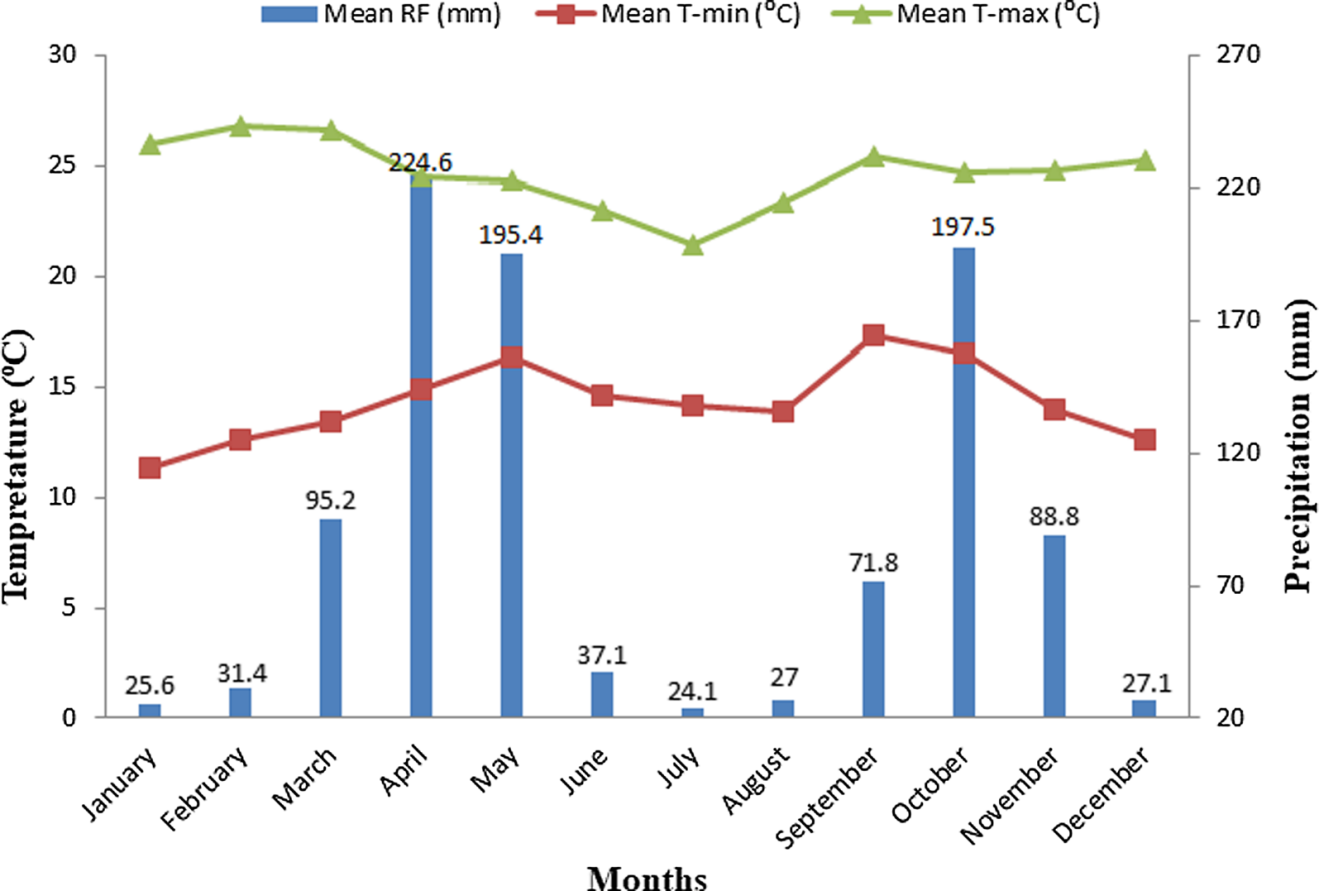
Climate diagram of Odo Shakiso district (Data Source: NMSA, 2017)

According to the climate data (1987–2017) obtained from the National Meteorological Services Agency (NMSA) [49], the area has a bimodal rainfall pattern and about 49.3 and 34.2% fall during summer (March–May) and autumn (September–November) seasons. Similarly, the mean monthly temperature for the last 30 years (1987–2017) ranges from 11.3 °C to 26.8 °C with an average of 24.7 °C (Fig. 2).

### Land use and land cover

Satellite images from 1988, 2008, and 2018 were used to classify to three land uses and analyse trends of deforestation in the area. Thematic Mapper (TM), Enhanced Thematic Mapper (ETM) and Landsat 8 of the three acquisition years (1988, 2008 and 2018) with less than 10% cloud cover were acquired from path 168 and 059 raw for February and January, respectively. It was so difficult to get cloud-free images in the other months in the study area for its bimodal rainfall pattern. Source of land use and land cover was freely downloaded as Landsat imagery from http://earthexplorer.usgs.gov/, and interviews and group discussions were conducted in the study to verify the accuracy of the classified images. The detail of the satellite data is presented in Table1. The imagery was processed using ENVI 5.0, and Arc GIS was used for slope generation. Pixels were clustered into categories of the forest, cultivated land and agroforestry.

**Table 1:** Area of LULC types during 1988, 2008 and 2018

LULC type	1988		2008		2018	
	Area (ha)	%	Area (ha)	%	Area (ha)	%
Agroforestry	1136	32	818.98	23.27	1575.4	44.8
Cultivated land	517	15	1235.99	35.12	955.1	27.1
Forest land	1866	53	1464.46	41.61	988.4	28.1
Total	3519	100	3519.43	100	3519	100

### Woody species surveys

100 plots placed at random intervals along transect were surveyed. The number of plots per land use type was approximately proportional to area covered by the land use. Based on reconnaissance survey piloted from 1 to 20 November 2018 and lulc analysis, the watershed was classified into three land uses to get impression of the watershed`s physiognomy. A systematic random sampling technique was employed to select study plots for collecting vegetation and environmental data. Once the first sample plot was randomly established, subsequent independent sampling plots were laid down along line transects at every 300 m intervals between each sampling plot. Line transects are 1 km apart from each other using a global positioning system (GPS). Quadrat sizes were determined using the minimal area method following [32, 47]. Sampling plots of 20 × 20 m^2^ were used for measuring woody species. The woody plant species in each sample quadrants were recorded and coded with vernacular and local names. Species specimens were collected, pressed, dried for taxonomic identification comparing with already identified specimens, experts’ consultation, and referring books at the National Herbarium, Addis Ababa University [17, 27]. In each quadrat, plants with a height of ≥ 3 m were considered as shrub or trees following [23].

### Data analysis

#### Soil sample analysis

[42] Found out that soil physicochemical variables were significantly affecting vegetation distribution in Afromontane. For analysing soil variables, soil samples up to 30 cm in depth with a soil auger were collected. 100 composite soil samples of the samples collected from four corners and one center of the quadrats were brought to soil laboratory at Batu Agricultural Research Center. The samples were dried at room temperature, ground, thoroughly mixed, and sieved through 2 mm mesh. The exchangeable cations (water suspension, soil to water ratio 1:2.5 using electro conductivity meter), available phosphorus, available potassium and cation exchange capacity (ammonium acetate method, 1MNH4OAc), total nitrogen (Kjeldahl method) [11], pH (water suspension, soil to water ratio 1:2.5 using pH meter), bulk density, organic carbon contents [28, 60].

### Woody species composition

Species diversity indices has emerged to assess conservation and ecological value of a site [39, 47]. Since Shannon wiener diversity index (H’) is not affected by sample size, diversity index of this study was calculated according to Shannon–Wiener [52]. Similarly, individual-based rarefaction was used to compare species richness for sample size varied among the land uses (Forest, Agroforestry, and Cultivated land), computed using ‘PAST’ version 3.06 [13].

Hierarchical cluster analysis was performed to identify communities based on floristic similarities using R-free statistical software version 3.4.1 [32, 50]. Dominant species of each community type were identified based on their synoptic values and community types were named after one or more dominant species [43]. Furthermore, the relationship between woody species community and environmental variables was analysed with the ordination program “Canonical Correspondence Analysis (CCA)” using log-transformed abundance data of the three land uses. The resulting ordination being a product of both variabilities of environment and species data, the diagram expresses patterns of variation in floristic composition and demonstrates the principal relationships between species and environmental variables [33, 64].

## Results

### Land use/ cover change for 1988, 2008 and 2018

**Fig. 3:**
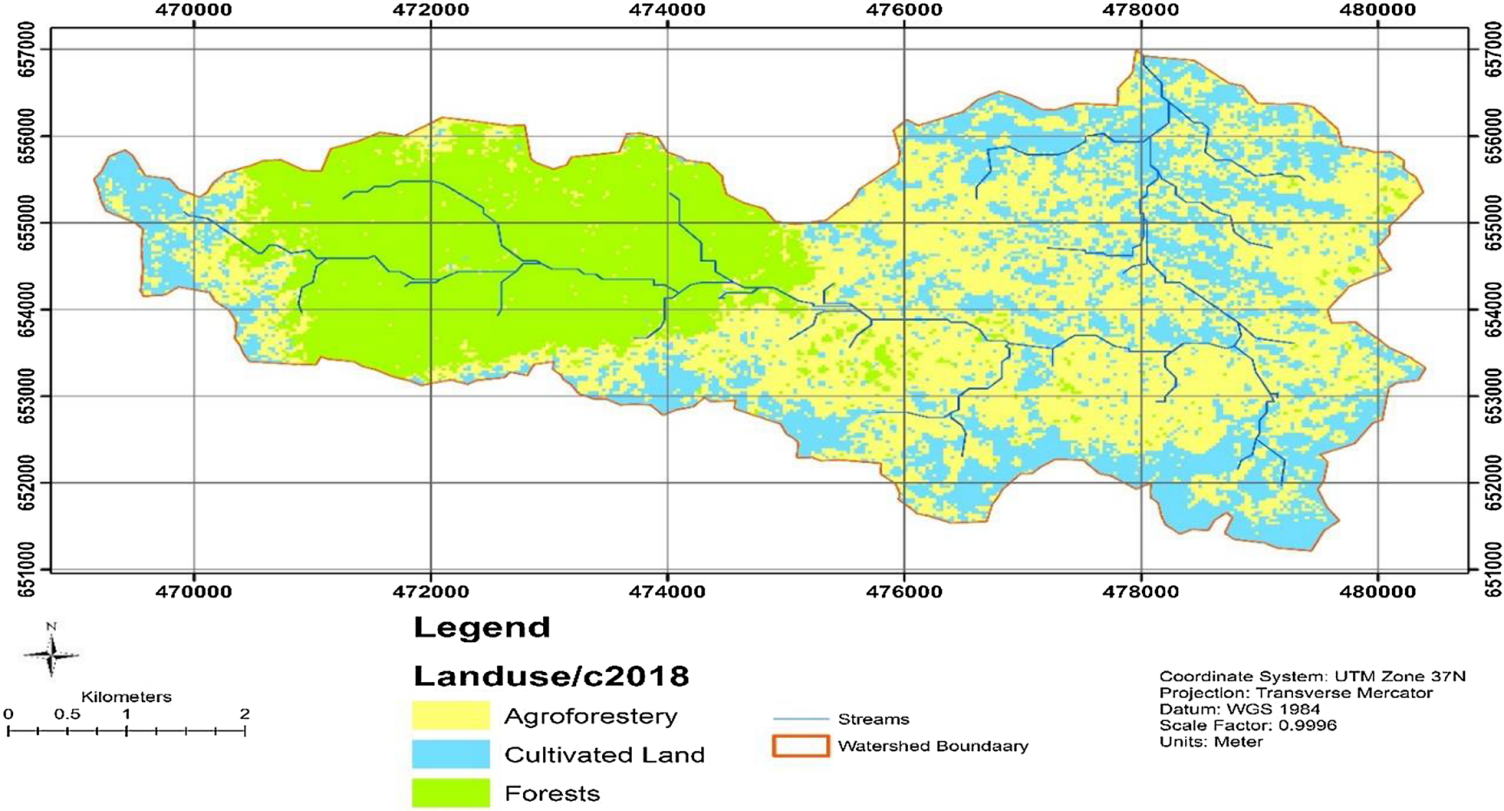
Land use land cover map of Hangadi watershed

The dominant land use/land cover classes in 1988 were forest and agroforestry with an area of 1866 ha (53%) and 1136 (32%). The least coverage was cultivated land, which accounted for 517 ha (15%). 85% of the study area was covered by green vegetation such as forest and agroforestry, while the remaining 15% was covered by cultivated land in 1988 (Table 1). Contrasting 1988, the dominant land use/land cover classes in 2008 were forest and cultivated land with an area of 1464. 46 ha (41.61%) and 1235. 99 ha (35.12%), respectively. Agroforestry had the least area coverage of about 818.98 ha (23.27%). 64.88% was covered by green vegetation such as forest and agroforestry; the remaining 35.12% was covered by cultivated land in 2008 (Table 1 and Fig. 3). In 2018, the dominant LULC class was found to be agroforestry covering an area of 1575.4 ha (44.8%) followed by forest land of 988.4 ha (28.1%), and the cultivated land accounted for 955.1 ha (27.1%). Over these periods in time, forest land was decreasing while agroforestry was increasing except in 2008.

### Woody species composition

**Fig. 4:**
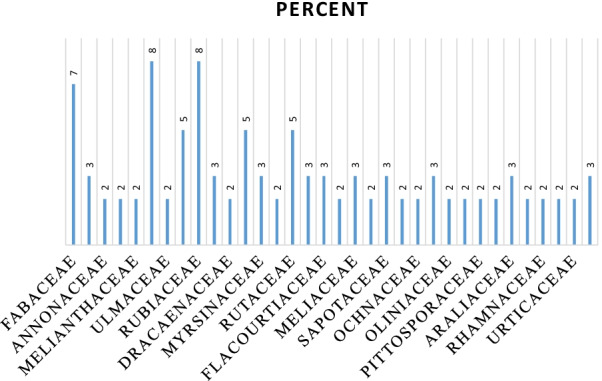
Percent Species contribution of the families in Hangadi watershed

A total of 61 woody species were yielded from the study representing 34 families in watershed. The highest species number was recorded from the families of Euphorbiaceae and Rubiaceae (5 species, 8.2%). Fabaceae (4 species, 6.78%), Rutaceae, Celastraceae, and Oleaceae (3 species, 5.08%) followed by Flacourtiaceae, Meliaceae, Araliaceae, Myrsinaceae, Moraceae, Boraginaceae, Asteraceae, Sapotaceae, Lauraceae, and Sapindaceae (2 species, 3.39% each). The families that contributed to the total woody species in the study area are represented (Fig. 4), and endemic species recorded and level of threat is presented.

**Fig. 5:**
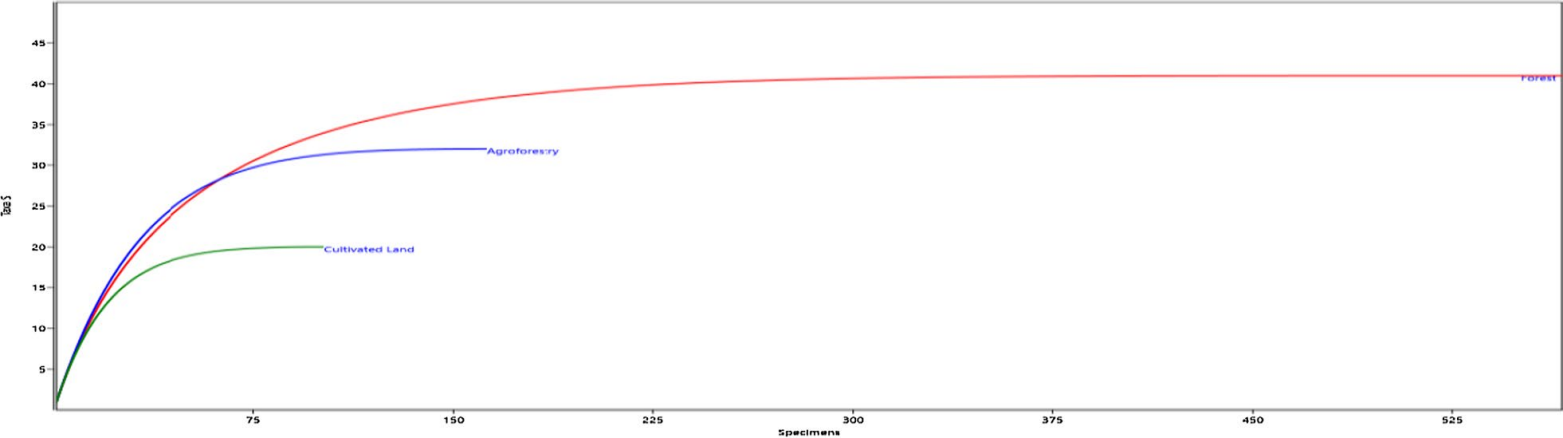
Species accumulation curve for Hangadi watershed

The Shannon–Wiener diversity index and evenness values in the study area were 3.6 and 0.89, respectively (Table 2). Individual-based rarefied richness showed there is variation among land-uses (Fig. 5). The three land-use types showed variation in their species richness. The forest land use type (F) had the highest species richness, diversity but the second highest in Simpson evenness next to cultivated land (C).

**Table 2:** Species Richness, Diversity and Evenness Values of Land Uses

LU	Richness	H	Simpson	Shannon-Evenness	Simpson-Evenness	Margalef	Hill
F	58	3.53	26.53	0.89	0.47	19.18	46.74
A	32	2.43	7.41	0.86	0.37	7.19	15.9
C	15	2.06	6.14	0.81	0.56	3.59	9.5

### Plant communities

**Fig. 6:**
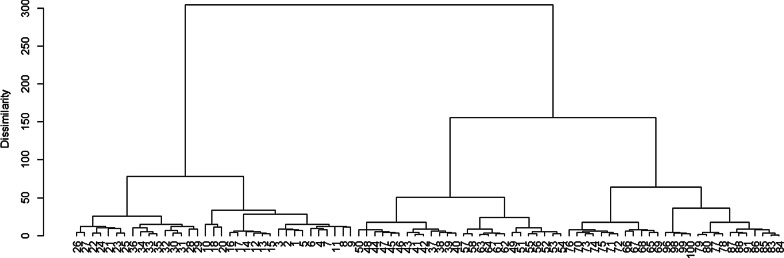
Dendrogram showing the relationship among the land uses

Four woody species community types were identified from hierarchical cluster analysis based on abundance data of the species on the study plots (Fig. 6). Two or more species were used to name the corresponding woody species communities and relationship was also indicated in Table 3.

**Table 3:** Species richness and diversity values of Community Types in Hangadi watershed

Community	Elevation	Species Richness	Shannon diversity	Evenness	Average slope (%)
1	1784–2167	28	2.9	0.87	15.6 (moderate)
2	1784–2165	59	3.56	0.89	20.17 (strong)
3	2013–2187	53	3.47	0.88	19.4 (strong)
4	2044–2100–2155	27	2.88	0.87	

Community 1: *Polyscias fulva* (Hiern) Harms—*Dracaena afromontana* Mildbr

The *Polyscias fulva* (Hiern) Harms—*Dracaena afromontana* Mildbr community found in the altitudinal distribution ranges from 1784 to 2167 m. a.s.l. *Allophylus abyssinicus* (Hochst.) Radlk, *Dracaena afromontana* Mildbr, *Embelia schimperi* Vatke, *Galiniera saxifraga* (Hochst.) Bridson, *Ilex mitis* (L.) Radlk, *Lepidotrichilia volkensii* (Gürke) Leroy, *Ochna holstii* Engl, *Pouteria adolfi-friederici* (Engl.) Baehni, *Psychotria orophila* Petit, *Psydrax schimperiana* (A. Rich.) Bridson, *Syzygium guineense* (Willd.) DC subsp. afromontanum F. White, *Teclea nobilis* Del., *Urera hypselodendron* (A. Rich.) Wedd, *Vepris dainellii* (Pic.Serm.) Kokwaro, *Polyscias fulva* (Hiern) Harms, *Elaeodendron buchananii* (Loes.) Loes, *Ocotea kenyensis* (Chiov.) Robyns & Wilczek, *Olea capensis* L. subsp. macrocarpa (C.A. Wright.) Verdc, *Bridelia micrantha* (Hochst.) Baill, *Erythrococca trichogyne* (Muell Arg.) Prain, *Fagaropsis angolensis* (Engl.) Dale, *Ficus thonningii* Blume, *Macaranga capensis* (Baill.) Sim, *Olea welwitschii* (Knobl.) Gilg & Schellenb, *Pavetta abyssinica* Fresen, *Apodytes dimidiata* E. Mey. ex Arn, *Oncoba spinosa* Forssk, *Croton macrostachyus* Del, *Deinbollia kilimandscharica* Taub, *Ficus sur* Forssk, *Pittosporum viridiflorum* Sims, *Podocarpus falcatus* (Thunb.) R. B. ex Mirb, *Rytigynia neglecta* (Hiern) Robyns, *Schefflera abyssinica* (Hochst. ex A. Rich.) Harms, *Rhamnus prinoides* L’Herit, *Annona senegalensis* Pers, *Coffea arabica* L, *Cordia africana* Lam, *Persea americana* Mill, *Trichilia emetica* Vahl, *Ensete ventricosum* (Welw.) Sheeseman, *Albizia gummifera* (J.F. Gmel.) C.A. Sm, *Chionanthus mildbraedii* (Gilg & Schellenb.) Stearn, *Nuxia congesta* R. Br. ex Fresen, *Olinia rochetiana* A. Juss, *Phytolacca dodecandra* L’Herit, *Prunus africana* (Hook. f.) Kalkm, *Vernonia amygdalina* Del, *Vernonia rueppellii* Sch. Bip. ex Walp, *Celtis africana* Burm. f, *Ehretia cymosa* Thonn, *Lobelia giberroa* Hemsl and *Maesa lanceolata* Forssk in which 60.4%, 34%, 3.8% and 1.8% tree, shrub, lianas and herb, respectively.

Community 2: *Croton macrostachyus* Del.—*Teclea nobilis* Del

This community type was found between 1784 and 2165 m.a.s.l. *Allophylus abyssinicus* (Hochst.) Radlk., *Cordia africana* Lam., *Deinbollia kilimandscharica* Taub, *Dracaena afromontana* Mildbr., *Ehretia cymosa* Thonn, *Erythrococca trichogyne* (Muell Arg.) Prain, *Ficus thonningii* Blume, *Hippocratea africana* (Willd.) Loes., *Ilex mitis* (L.) Radlk., *Lepidotrichilia volkensii* (Gürke) Leroy, *Lobelia giberroa* Hemsl., *Macaranga capensis* (Baill.) Sim, *Maesa lanceolata* Forssk., *Maytenus arbutifolia* (A. Rich.) Wilczek, *Millettia ferruginea* (Hochst.) Bak. subsp. darassana (Cuf.) Gillett, *Mimusops kummel* A. DC., *Olea capensis* L. subsp. macrocarpa (C.A. Wright.) Verdc., *Olinia rochetiana* A. Juss, *Pavetta abyssinica* Fresen., *Phytolacca dodecandra* L ’Herit., *Pittosporum viridiflorum* Sims*, Polyscias fulva* (Hiern) Harms, *Pouteria adolfi-friederici* (Engl.) Baehni, *Prunus africana* (Hook. f.) Kalkm., *Psydrax schimperiana* (A. Rich.) Bridson, *Rytigynia neglecta* (Hiern) Robyns, *Schefflera abyssinica* (Hochst. ex A. Rich.) Harms, *Syzygium guineense* (Willd.) DC subsp. afromontanum F. White, *Teclea nobilis* Del., *Trichilia emetica* Vahl, *Vernonia amygdalina* Del., *Vernonia rueppellii* Sch. Bip. ex Walp, *Elaeodendron buchananii* (Loes.) Loes., *Nuxia congesta* R. Br. ex Fresen., *Ocotea kenyensis* (Chiov.) Robyns & Wilczek, *Podocarpus falcatus* (Thunb.) R. B. ex Mirb., *Celtis africana* Burm. f., *Coffea arabica* L., *Ensete ventricosum* (Welw.) Sheeseman, *Persea americana* Mill., *Croton macrostachyus* Del., *Bersama abyssinica* Fresen., *Vepris dainellii* (Pic.Serm.) Kokwaro, *Apodytes dimidiata* E. Mey. ex Arn., *Embelia schimperi* Vatke, *Erythrina brucei* Schweinf., *Oncoba spinosa* Forssk, *Urera hypselodendron* (A. Rich.) Wedd., *Calpurnia aurea* (Ait.) Benth., *Fagaropsis angolensis* (Engl.) Dale, *Flacourtia indica* (Burm. f) Merr., *Euphorbia abyssinica* J.F. Gmel., *Galiniera saxifraga* (Hochst.) Bridson, *Psychotria orophila* Petit, *Bridelia micrantha* (Hochst.) Baill., *Chionanthus mildbraedii* (Gilg & Schellenb.) Stearn, *Olea welwitschii* (Knobl.) Gilg & Schellenb., *Ficus sur* Forssk., and *Rhamnus prinoides* L’Herit., of which 1.7% is a herb, *Ensete ventricosum* (Welw.) Sheeseman.

Community 3: *Ensete ventricosum* (Welw.) Sheeseman—*Coffea arabica* L

This community type was distributed in the altitudinal range between 2013 and 2187 m.a.s.l. *Coffea arabica* L., *Croton macrostachyus* Del., *Erythrina brucei* Schweinf, *Euphorbia abyssinica* J.F. Gmel., *Podocarpus falcatus* (Thunb.) R. B. ex Mirb., *Pouteria adolfi-friederici* (Engl.) Baehni, *Allophylus abyssinicus* (Hochst.) Radlk., *Ensete ventricosum* (Welw.) Sheeseman, *Millettia ferruginea* (Hochst.) Bak. subsp. darassana (Cuf.) Gillett, *Vernonia amygdalina* Del., *Prunus africana* (Hook. f.) Kalkm., *Schefflera abyssinica* (Hochst. ex A. Rich.) Harms, *Vernonia rueppellii* Sch. Bip. ex Walp., *Dracaena afromontana* Mildbr., *Bersama abyssinica* Fresen., *Elaeodendron buchananii* (Loes.) Loes., *Ficus thonningii* Blume, *Trichilia emetica* Vahl, *Ehretia cymosa* Thonn., *Albizia gummifera* (J.F. Gmel.) C.A. Sm, *Flacourtia indica* (Burm. f) Merr., *Polyscias fulva* (Hiern) Harms, *Fagaropsis angolensis* (Engl.) Dale, *Calpurnia aurea* (Ait.) Benth., *Deinbollia kilimandscharica* Taub, *Lobelia giberroa* Hemsl., *Olea capensis* L. subsp. macrocarpa (C.A. Wright.) Verdc. and *Celtis africana* Burm. f., 3.6% is attributed to herb, Ensete ventricosum (Welw.) Sheeseman.

Community 4: Olea capensis L. subsp. macrocarpa (C.A. Wright.) Verdc.—Coffea arabica L.—Pouteria adolfi-friederici (Engl.) Baehni

Altitudinal distribution of this community ranges from 2044, 2100 and 2155 m.a.s.l. *Calpurnia aurea* (Ait.) Benth., *Coffea arabica* L., *Deinbollia kilimandscharica* Taub, *Euphorbia abyssinica* J.F. Gmel., *Fagaropsis angolensis* (Engl.) Dale, *Polyscias fulva* (Hiern) Harms, *Celtis africana* Burm. f., *Olea capensis* L. subsp. macrocarpa (C.A. Wright.) Verdc., *Prunus africana* (Hook. f.) Kalkm., *Millettia ferruginea* (Hochst.) Bak. subsp. darassana (Cuf.) Gillett, *Annona senegalensis* Pers., *Cordia africana* Lam., *Elaeodendron buchananii* (Loes.) Loes., *Ocotea kenyensis* (Chiov.) Robyns & Wilczek, *Persea americana* Mill., *Podocarpus falcatus* (Thunb.) R. B. ex Mirb., *Trichilia emetica* Vahl, *Albizia gummifera* (J.F. Gmel.) C.A. Sm., *Croton macrostachyus* Del., *Dracaena afromontana* Mildbr., *Ehretia cymosa* Thonn., Ficus sur Forssk., *Pouteria adolfi-friederici* (Engl.) Baehni, *Ensete ventricosum* (Welw.) Sheeseman, *Lobelia giberroa* Hemsl., *Flacourtia indica* (Burm. f) Merr., *Syzygium guineense* (Willd.) DC subsp. afromontanum F. White and *Erythrina brucei* Schweinf., 78.5%, 17.9%, and 3.6% are attributed by tree, shrub, and herb, respectively.

**Fig. 7:**
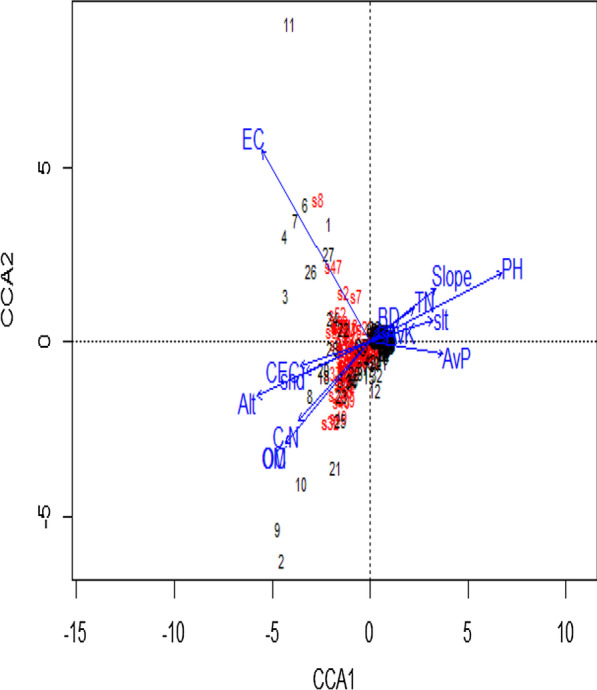
CCA of sites constrained by some environmental variables and community types in three land uses of Hangadi watershed

CCA examination revealed that statistically significant differences were observed among the species composition and environmental variables collected from this study. A selection procedure screened out the following environmental variables (Table 4) to be more responsible (P < 0.005) for the distribution of woody species and their community composition in the study area. The four community types of the Hangadi watershed showed variation in their species richness, diversity and evenness. This variation among community types was a direct reflection of the effects of the environmental variables where these community types occurred. Comparatively community three was the most diverse and richest in its species composition than other communities; might be attributed to the combined effect of C: N, AvK, pH, slope, CEC, altitude, silt, and BD in the case of the forest, and EC, pH, OM, altitude, C: N, CEC, sand, silt, slope, AvP and TN in the three land uses of the watershed. Generally, this might be attributed to combined effects of the edaphic and topographic differences (Fig. 7).

**Table 4:** Vif values in Hangadi watershed

Variables	Vif	Rank	CCA
EC	1.00	1	2
pH	1.18	2	1
OM	1.50	3	2
Altitude	1.97	4	1
C:N	2.24	5	2
CEC	2.50	6	1
Sand	2.60	7	1
Slope	2.60	8	1
Silt	2.79	9	1
AvP	3.80	10	1
TN	4.00	11	1

### Important Value Index (IVI)

In Hangadi watershed, the highest IVI value was recorded for Ensete ventricosum (Welw.) Sheeseman (18), followed by Coffea arabica L (17.1), Dracaena afromontana Mildbr. (14), Podocarpus falcatus (Thunb.) R. B. ex Mirb (8.9), Syzygium guineense (Willd.) DC subsp. afromontanum F. White (9.6), Pouteria adolfi-friederici (Engl.) Baehni (9), Polyscias fulva (Hiern) Harms (10), Olea capensis L. subsp. macrocarpa (C.A. Wright.) Verdc. (10), Prunus africana (Hook. f.) Kalkm. (9), Croton macrostachyus Del. (10) and Ocotea kenyensis (Chiov.) Robyns & Wilczek (8). These eleven species contributed about 41.2% of the total importance values whereas the remaining 51 species had combined IVI values of 58.8% (Table 3).

## Discussion

### Woody species composition

Hangadi watershed is home to a diversity of 61 woody species. The number of woody species recorded was higher than for similar forest type in different parts of the country. For example, the Agama tropical Afromontane forest had 39 woody species [1]. Tadele et al. [53] also recorded a lower number of species (50 species) in Zengena Forest in Ethiopia. Moreover, [5] recorded 44 woody species in Doshke forest, Chincha. On the other hand, [8, 10, 31, 58, 63] recorded a higher woody species of 64, 66, 72, 143 and 74 in Beseku, Kuandisha, Wondo Genet, Tera Gedam and Sirso Afromontane forests, respectively. In Ethiopia, the available floristic data are either site-specific [e.g., 53] or covering a wide range of vegetation types [21], as a result, it is difficult to make a direct comparison with other similar studies. The reasons for variation in floristic composition at the study sites could be due to excessive anthropogenic disturbances and land exploitation of some species (for instance community 1) and several environmental factors that operate over multiple temporal and spatial scales [7, 34]. Climate and topography appear to have broad effects on diversity across the landscape, while biological factors and availability of suitable environmental gradients seem to influence diversity more at the site level [44].

The family Euphorbiaceae and Rubiaceae have the highest representation of species (5 species, 8.2%) each in Hangadi watershed. [59] stated that Euphorbiaceae was among the richest family in the flora area (Flora of Ethiopia and Eritrea). This could also be related to its efficient and successful dispersal strategies as well as better adaptation to a wide range of ecological conditions. Euphorbiaceae was also found to be one of the dominant families in other Afromontane forests in Ethiopia like Komto (8 species, 4.44%) [20], Jibat (7 species, 4.4%) [57], Gratkhassu and Hugumburda (7species, 3.1%; 7 species, 3.3%, respectively [36]. However, in terms of species richness found in the FEE, Fabaceae (678 species), Poaceae (609 species), Asteraceae (472 species) and Euphorbiaceae (472 species) were the richest families [18].

The number of endemic species harboured in the watershed was 5 species. Though the forest is relatively poor in diversity of woody plant species endemic to Ethiopia, it is better than for instance, the yayu forest with three endemic plant species [55]. The proportion of endemic plant species in other Afromontane forests of Ethiopia is high, ranging between 11 and 15% of the total number of species [21]. 8.7% (31 species) of the plant species found in Borena Saint National Park were endemic [28]. In agreement with this study, southwest moist montane forests are poor in trees/shrubs endemicity compared to dry Afromontane forests [35]. It has been known that the Afromontane forests contained higher endemism than the other regions in Africa. Over 3000 endemic plant species are estimated to be found in this region [61]. However, there has been a threat to the endemic plants found in the study area and thus need immediate action to protect them. All the recorded endemic woody species found in the Hangadi watershed are already on the Red List of endemic species of Ethiopia and Eritrea. Erythrina brucei, Millettia ferruginea, Vepris dainelli, and Vernonia ruepelli were species with the least concern while Maytenus addat is near-threatening [59].

### Woody species community types and diversity

Three land-uses of the watershed showed variation in their species richness, diversity, and evenness. Variation among the land uses was direct reflection of the effects of environmental variables. Comparatively, forest land use was the most diverse and the richest in its species composition than the other land uses. It might be attributed to the combined effects of anthropogenic and edaphic differences, as the representative plots were composed of three land-use types (Forest, Agroforestry, and cultivated land) and the concomitant occurrence of species adapted to these different environmental conditions. The difference in terrain, soils, water, and microclimatic conditions cause differences in species adaptability [2, 4].

The Shannon Wiener diversity index was high (H’ = 3.6) in the study area. It normally varies between 1.5 and 3.5, rarely exceeds 4.5 whose value is found to be higher than that of other montane forests, such as Chilimo (H’ = 2.72; [56], Tara Gedam (H’ = 2.98; [26]. The difference in Shannon diversity index between forest land use (F = 3.6) and cultivated land (C = 2.06) might be related to higher anthropogenic disturbances in the cultivated land. According to field observation and focal group discussion, cultivated land was heavily affected by the local people involved in clearing forests for the expansion of the farmland already at their hand to cultivate cereals, pulse, and perennial crops. Moreover, habitat diversity is a widely accepted determinant of species diversity [48].

### Woody species community- environmental variables relationship

While comparing the land uses (Table 3) and (Fig. 3 and Table 4), the community types of the watershed showed variation in richness and diversity. The variation among the community types could be the effects of the environmental variables. Community 2 was the most diverse and richest in species composition comparing with the other communities. This might be attributed to the effects of topographic and edaphic differences and the concomitant occurrence of species adapted to different environmental conditions.

Community 2 and 3 are the first and the second both in species richness and Shannon wiener diversity. This could be associated with having intermediate or reduced disturbances since most of the plots of these two communities were found on the sloppy area (20.17 and 19.40), respectively. The two communities are composed of plots of forest and agroforestry land-uses. The coffee agroforestry plots are RFA (Rainforest Alliance) certified with high value conservation areas whereby no crop is cultivated, intended only for a biodiversity conservation. Similar, results were indicated by [44] explaining that intermediate levels of forest disturbance may promote community diversity by facilitating regeneration of some species.

The relationship between plant communities, soil properties and topography is important in understanding the woody species communities in a given ecosystem [7, 37]. Our results show spatial variability in soil characteristics and topography across the watershed significantly affected distribution of woody plant species among the identified communities. The dominant woody species in the study watershed are Dracaena afromontana, Teclea nobilis, Ocotea kenyensis, Syzygium guineense subsp. Afromontanum, Olea capensis subsp. macrocarpa. Pouteria adolfi-friederici, Psychotria orophila, Croton macrostachyus, Polyscias fulva. A similar pattern was reported as the characteristic of the Afromontane rainforest [21].

CCA was used to evaluate distribution pattern of 61 woody plant species influenced by environmental variables. Of the environmental variables: EC, pH, OM, altitude, C:N, CEC, sand, slope, silt, AvP and TN significantly influenced the species distribution. Community diversity plays a major role in ecology and conservation biology since it is an important parameter of a plant community concerning ecosystem dynamics and environmental quality [40]. While comparing the land uses (Table 3) among each other (Fig. 4 and Table 4), the community types of the watershed showed variation in richness and diversity. The variation among the community types could be the effects of the environmental variables. Community 2 was the most diverse and richest in species composition comparing with the other communities. This might be attributed to the effects of topographic and edaphic differences and the concomitant occurrence of species adapted to these different environmental conditions.

There is a difference in the Shannon wiener diversity index in the study area. The difference in the Shannon diversity index among communities (Community 1; H’ = 2.9), (community 2; H’ = 3.4), and (community 3; H’ = 3.48) might be related to high anthropogenic disturbances in the community 1. According to the focal group discussion and field observation, community 1 was heavily affected by the local people involved in the cutting of mature woody species for timber production, for making farm implements and beehive, charcoal production, cultivated land expansion, and house construction. Local climatic variations and forest disturbances are mentioned among the factors most responsible for variations in species diversity in a given forest due to their effect on the removal of some preferred species, and the resulting change in the light environment of the understorey species [3].

Community 2 and 3 are the first and second, both in species richness and Shannon wiener diversity; could be associated with intermediate or reduced disturbances since most of the plots of these two communities were found on the sloppy area (20.17 and 19.4), respectively. These two communities could not be easily accessible by the local people to exploit through selective cutting and grazing animals; similar results were reported by [5] explaining forest community diversity is affected by the slope of the area.

### Importance Value Index

The greatest IVI reflects the extent of dominancy in a given species in comparison to other species in the structure of a forest stand. According to [41], species with the highest importance value index are the most dominant of the particular vegetation. It is also used for setting priority species management and conservation practices [17].

For the sake of setting species priority for conservation using IVI analysis, all woody plant species encountered in the forest were grouped into three IVI classes based on their total IVI values (Tables 5, 6, 7). Accordingly, 2 woody species are found to be with less than 1, 57 species with 1–10, and 3 species with 10–20 IVI values. Those species that exhibit lower IVI values need high conservation efforts while those with higher IVI values need monitoring management [45].

**Table 5:** Importance Value Index (IVI) table based on: IVI = Relative Density (RD) + Relative Dominance (RDO) + Relative Frequency (RF)

Species name	Habitat and common use	RD	RDO	RF	IVI
Albizia gummifera (J.F. Gmel.) C.A. Sm	T t	0.6	1.5	2.5	4.6
Allophylus abyssinicus (Hochst.) Radlk	T t	3	1.3	1.8	6.2
Annona senegalensis Pers	T t	0.3	0.3	0.7	1.3
Apodytes dimidiata E. Mey. ex Arn	T t	3.2	0.6	0.7	4.5
Bersama abyssinica Fresen	T m	0.5	0.6	1	2.1
Bridelia micrantha (Hochst.) Baill	T fm	1.3	0.3	0.7	2.3
Calpurnia aurea (Ait.) Benth	S m	0.6	1.3	1.5	3.5
Celtis africana Burm. f	T t	2.8	1.4	2.2	6.4
Chionanthus mildbraedii (Gilg & Schellenb.) Stearn	S m	1.2	0.3	0.3	1.8
Coffea arabica L	S fm	0.3	11.6	5.2	17.1
Cordia africana Lam	T t	4	0.7	1	5.7
Croton macrostachyus Del	T t	3	2.2	4.5	10
Deinbollia kilimandscharica Taub	S f	0.5	0.8	1	2.3
Dracaena afromontana Mildbr	T o	2	7.7	4.2	14
Ehretia cymosa Thonn	T t	1	0.7	1	2.7
Elaeodendron buchananii (Loes.) Loes	T m	2	2	2	7
Embelia schimperi Vatke	L m	1	1	1.2	3.1
Ensete ventricosum (Welw.) Sheeseman	H f	4	9.1	4.5	18
Erythrina brucei Schweinf	T m	1	0.8	0.8	2.6
Erythrococca trichogyne (Muell Arg.) Prain	S m	0.6	0.4	0.5	1.5
Euphorbia abyssinica J.F.Gmel	T t	3	1.8	2.7	7.5
Fagaropsis angolensis (Engl.) Dale	T t	0.9	1	1.3	3.3
Ficus sur Forssk	T t	5.9	0.5	0.7	7.1
Ficus thonningii Blume	S m	0.9	0.4	0.8	2.2
Flacourtia indica (Burm. f) Merr	S tf	0.8	1	1.3	3.1
Galiniera saxifraga (Hochst.) Bridson	S t	1.2	0.6	0.5	2.3
Hippocratea africana (Willd.) Loes	L m	0.5	0.2	0.3	1
Ilex mitis (L.) Radlk	T tm	2	0.1	0.7	2.8
Lepidotrichilia volkensii (Gürke) Leroy	T tf	0.9	2.4	1.8	5.1
Lobelia giberroa Hemsl	S m	1	1	1	2
Macaranga capensis (Baill.) Sim	T m	3	0.5	0.7	4.2
Maesa lanceolata Forssk	T t	0.3	0.7	0.5	1.5
Maytenus addat (Loes.) Sebsebe	S F	1	1	1	3
Millettia ferruginea (Hochst.) Bak. subsp. darassana (Cuf.) Gillett	T t	1	2.8	1.8	5.6
Mimusops kummel A. DC	T t	0.5	0.2	0.3	1
Nuxia congesta R. Br. ex Fresen	T m	1.3	0.5	0.7	2.5
Ochna holstii Engl	S m	0.4	0.2	0.3	0.9
Ocotea kenyensis (Chiov.) Robyns & Wilczek	T t	3	2	3	8
Olea capensis L. subsp. macrocarpa (C.A. Wright.) Verdc	T t	3	3	4	10
Olea welwitschii (Knobl.) Gilg & Schellenb	T t	1	1	1	2
Olinia rochetiana A. Juss	T t	0.4	0.2	0.3	0.9
Oncoba spinosa Forssk	S t	0.3	0.5	0.5	1.3
Pavetta abyssinica Fresen	S m	0.5	0.6	0.7	1.8
Persea americana Mill	T f	0.1	1	1	2.1
Phytolacca dodecandra L 'Herit	S m	1.1	0.2	0.3	1.6
Pittosporum viridiflorum Sims	T t	3	1	1	5.1
Podocarpus falcatus (Thunb.) R. B. ex Mirb	T t	2	3.3	3.6	8.9
Polyscias fulva (Hiern) Harms	T m	3	2.5	4	10
Pouteria adolfi-friederici (Engl.) Baehni	T t	3	3.2	3.2	9
Prunus africana (Hook. f.) Kalkm	T t	3	2	4	9
Psychotria orophila Petit	S f	3.1	1.1	1.2	5.4
Psydrax schimperiana (A. Rich.) Bridson	T m	1	2	2	5
Rhamnus prinoides L’Herit	S m	1	0.1	1	2
Rytigynia neglecta (Hiern) Robyns	S f	1	1	1	3
Schefflera abyssinica (Hochst. ex A. Rich.) Harms	T t	2	1.8	2.2	6
Syzygium guineense (Willd.) DC subsp. afromontanum F. White	T t	4	2.4	3.2	9.6
Teclea nobilis Del	S t	1.2	3.5	2.3	7
Trichilia emetica Vahl	T t	1.3	0.8	1.2	3.3
Urera hypselodendron (A. Rich.) Wedd	L f	1	1	1	3
Vepris dainellii (Pic.Serm.) Kokwaro	S t	0.9	2.3	1.4	4.6
Vernonia amygdalina Del	S m	1	2	2	5
Vernonia rueppellii Sch. Bip. ex Walp	S m	1.6	2	1.2	4.8
Total		100	100	100	300

*Tt* tree-timber, *Tm* tree-medicine, *Tfm* tree-food-medicine, *sm* shrub-medicine, *sfm* shrub-food-medicine, *sf* shrub-food, *To* tree-ornamental, *Lm* Liana-medicine, *Hf* herbal-food, *stf* shrub-timber-food, *Ttm* tree-timber-medicine, *Ttf* tree-timber-food, *st* shrub-timber

**Table 6:** IVI classes and the number of species belonged to each class

IVI class and values	Number of species	Sum of IVI	Percentage
5 (< 1)	2		3.2
4 (1–10)	57		91.9
3 (10.0–20)	3		4.9

**Table 7:** List of species under each IVI Priority Class

Priority class		
5	4	3
Ochna holstii Engl, Olinia rochetiana A. Juss. Albizia gummifera (J.F Gmel.) C.A. Sm Oncoba spinose Forssk		Coffea arabica L., Ensete ventricosum (Welw.) Sheeseman
	Trichilia emetica Vahl	
	Erythrina brucei Schweinf	
	Apodytes dimidiata E. Mey. ex Arn	
	Fagaropsis angolensis (Engl.) Dale	
	Urera hypselodendron (A. Rich.) Wedd	
	Polyscias fulva (Hiern) Harms	
	Embelia schimperi Vatke	
	Ficus thonningii Blume	
	Cordia africana Lam	
	Hippocratea africana (Willd.) Loes	
	Erythrococca trichogyne (Muell Arg.) Prain	
	Phytolacca dodecandra L ’Herit	
	Macaranga capensis (Baill.) Sim	
	Vernonia rueppellii Sch. Bip. ex Walp	
	Lobelia giberroa Hemsl	
	Pouteria adolfi-friederici (Engl.) Baehni	
	Rytigynia neglecta (Hiern) Robyns	
	Pavetta abyssinica Fresen	
	Schefflera abyssinica (Hochst. ex A. Rich.) Harms Chionanthus mildbraedii (Gilg & Schellenb.) Stearn Persea americana Mill	
	Bersama abyssinica Fresen	
	Deinbollia kilimandscharica.Taub	
	Euphorbia abyssinica J.F.Gmel	
	Flacourtia indica (Burm. f) Merr	
	Psydrax schimperiana (A. Rich.) Bridson	
	Olea welwitschii (Knobl.) Gilg & Schellenb	
	Mimusops kummel A. DC	
	Vernonia amygdalina Del	
	Allophylus abyssinicus (Hochst.) Radlk	
	Galiniera saxifraga (Hochst.) Bridson	
	Celtis africana Burm. f	
	Calpurnia aurea (Ait.) Benth	
	Ehretia cymosa Thonn	
	Syzygium guineense (Willd.) DC subsp. afromontanum F. White Ilex mitis (L.) Radlk	
	Pittosporum viridiflorum Sims Olea capensis L. subsp. macrocarpa (C.A. Wright.) Verdc	
	Dracaena afromontana Mildbr Millettia ferruginea (Hochst.) Bak. subsp. darassana (Cuf.) Gillett	
	Maytenus addat (Loes.) Sebsebe	
	Bridelia micrantha (Hochst.) Baill	
	Rhamnus prinoides L’Herit	
	Croton macrostachyus Del	
	Prunus africana (Hook. f.) Kalkm	
	Maesa lanceolata Forssk	
	Psychotria orophila Petit	
	Teclea nobilis Del	
	Nuxia congesta R. Br. ex Fresen	
	Ficus sur Forssk	
	Lepidotrichilia volkensii (Gürke) Leroy	
	Vepris dainellii (Pic.Serm.) Kokwaro	
	Ocotea kenyensis (Chiov.) Robyns & Wilczek	
	Podocarpus falcatus (Thunb.) R. B. ex Mirb	
	Elaeodendron buchananii (Loes.) Loes	

## Conclusion

Hangadi watershed is one of the remnant vegetation in the Guji zone, southeastern Ethiopia. It constitutes a considerable number of woody plant species of high diversity, composition, and richness with 61 recorded species and four community types, which attributed to the availability of edaphic and topographic gradients that suits different woody plant associations. The four community types: Dracaena afromontana mildbr—Teclea noblis Del., Ocotea kenyensis (chiove.) Robyns (engl.) Baehni—Psychotria orophila petit, Olea capensis L.subsp.macrocarpa F. white, and Polyscias fulve (Hiern) Harms—Dracaena afromontana Mildbr, Croton macrostachyus Del—Teclea noblis Del, Enset ventricosum (welw.) sheesaman—Coffea arabica L., Olea capensis L. subsp. macracarpa (C.A. wright.) verdc—Coffea Arabica L.—Pouteria adolfi friderici (Engl.) Baehniin both forest and land use clusters, respectively (Figs. 4 and 5). Community 2 and 3 are the first and second in species richness and Shannon wiener diversity among the four community types, attributed to reduced disturbances for the majority of the plots of these communities were relatively found in the sloppy area. The distribution of woody species in the communities was commonly influenced by the edaphic variables (C: N, EC, CEC, pH, silt), and topographic gradients (altitude and slope). Local people heavily involved in cutting mature woody species for timber production, making farm implements and beehives, charcoal production, cultivated land expansion. The RFA certification with high value conservation plots in a farmer’s field whereby no crop is cultivated, meant only for a biodiversity conservation is recommended. Moreover, protection of the original woody species diversity of forest and profitable use of coffee systems with lower biodiversity value conservation concepts are recommended to be executed jointly by local people and other stakeholders prioritizing woody species with lower IVI values in the watershed.

## Data Availability

Not applicable. (The datasets used and/or analysed during the current study are available from the corresponding author on reasonable request.)
